# Promotion of Remyelination by Adipose Mesenchymal Stem Cell Transplantation in A Cuprizone Model of Multiple Sclerosis

**Published:** 2013-07-02

**Authors:** Azim Hedayatpour, Iraj Ragerdi, Parichehr Pasbakhsh, Laya Kafami, Nader Atlasi, Vahid Pirhajati Mahabadi, Soudabeh Ghasemi, Mahmoudi Reza

**Affiliations:** 1 Department of Anatomical Sciences, School of Medicine, Tehran University of Medical Sciences, Tehran, Iran; 2 Department of Pathobiology, School of Medicine, Alborz University of Medical Sciences, Karaj, Iran; 3 Department of Cellular and Molecular Research Center,Yasuj University of Medical Sciences,Yasuj, Iran

**Keywords:** Adipose, Mesenchymal Stem Cells, Demyelination, Transplantation

## Abstract

**Objective:**

Multiple sclerosis (MS) is an immune-mediated demyelinating disease of the central nervous system (CNS). Stem cell transplantation is a new therapeutic approach for demyelinating diseases such as MS which may promote remyelination. In this study, we evaluate the remyelinating potential of adipose mesenchymal stem cells (ADSCs) and their effect on neural cell composition in the corpus callosum in an experimental model of MS.

**Materials and Methods:**

This experimental study used adult male C57BL/6 mice. Cultured ADSCs were confirmed to be CD73^+^,CD90^+^, CD31^-^,CD45^-^, and labeled by PKH26. Animals were fed with 0.2% w/w cuprizone added to ground breeder chow ad libitum for six weeks. At day 0 after cuprizone removal, mice were randomly divided into two groups: the ADSCs-transplanted group and the control vehicle group (received medium alone). Some mice of the same age were fed with their normal diet to serve as healthy control group. Homing of ADSCs in demyelinated lesions was examined by fluorescent microscope. At ten days after transplantation, the mice were euthanized and their cells analyzed by luxol fast blue staining (LFB), transmission electron microscopy and flow cytometry. Results were analyzed by one-way analysis of variance (ANOVA).

**Results:**

According to fluorescent cell labeling, transplanted ADSCs appeared to survive and exhibited homing specificity. LFB staining and transmission electron microscope evaluation revealed enhanced remyelination in the transplanted group compared to the control vehicle group. Flow cytometry analysis showedan increase in Olig2 and O4 cells and a decrease in GFAP and Iba-1 cells in the transplanted group.

**Conclusion:**

Our results indicate that ADSCs may provide a feasible, practical way for remyelination in diseases such as MS.

## Introduction

Multiple sclerosis (MS) is a chronic demyelinating disease of the central nervous system (CNS) (1). The etiology and pathogenesis of MS are not completely understood and the treatments for MS are limited (2). Although several laboratory treatments have been shown to modify the course of MS, there is still no treatment that could halt or reverse the neurodegeneration that results from MS (1-3). The pathologic characteristics and treatment status make MS a good target for cell therapy. Several studies have shown the therapeutically benefit of neural stem cells (1), embryonic stem cells (2) and bone marrow mesenchymal stem cells (BMSCs) (3) in animal models of MS. However, embryonic stem cells can form teratomas (4) and the source of human neural stem cells is limited (5). Although BMSCs are available from the bone marrow of adults, they are limited in number andonly a few can be harvested from an individual (6).

Recently, much attention has been paid to adipose mesenchymal stem cells (ADSCs) because the adipose tissue is an abundant, easily accessible, and appealing source of donor tissue for cell transplantation (7). Some reports have recently demonstrated that ADSCs express α4 integrin (5, 8). *in vivo* studies stress the role of α4 integrin in influencing cell migration. Studies have begun to evaluate the clinical efficacy of using intravenously administered mesenchymal stem cells (MSCs) in diseases such as MS (5, 9). Several studies have demonstrated the migration of stem cells after intravenous injection (5, 10) inanimal models of MS. In this study we investigate the remyelination potential of intravenously transplanted ADSCs into demyelinated corpus callosum and their effect on neural cell composition in the corpus callosum.

## Materials and Methods

### Isolation of adiposemesenchymal stem cells

Epididymal fat pads of 8-week-oldmale C57BL/6 mice were excised, placed on a sterile glass surface, and finely minced. The minced tissue was placed in a 50 ml conical tube (Greiner, Germany) that contained 0.05% collagenase type 1 (Sigma, USA) and 5% bovine serum albumin (Sigma, USA). The tube was incubated at 37˚C for 1 hour. The tube contents, after filtering through a sterile 250 μm nylon mesh, were centrifuged at 250 g for 5 minutes. The cell pellet was resuspended in ADSC medium that consisted of Dulbecco’s modified eagle’s medium (DMEM; Gibco, USA), 10% fetal bovine serum (Gibco, USA), penicillin (100 U/ml), and 100 μg/ml of streptomycin (Sigma, USA). Cell count was determined with a hemacytometer (6).

Characterization of isolated adipose mesenchymal stem cells by flow cytometry We harvested mice ADSCs within 3-5 passages after the initial plating of the primary culture by trypsinization. The 10×10^5^ cells were fixed in a neutralized 2% paraformaldehyde (PFA) solution for 30 minutes. The fixed cells were washed twice with PBS and incubated with antibodies to the following antigens: CD31 (1:300), CD45 (1:300), CD73 (1:300) and CD90 (1:500; Chemicon, CA) for 30 minutes. Primary antibodies were directly conjugated with fluorescein isothiocyanate (FITC). The cells stained with FITC rat anti-mouse IgG served as controls. The specific fluorescence of 10000 cells was analyzed on a FACSCalibur (Becton Dickinson, USA) using Cell Quest Pro software (8).

### Homing assay

The homing efficiency of transplanted ADSCs was assayed by labeling ADSCs with the red fluorescent dye, PKH26, according to the manufacturer’s instructions (9). Animals were sacrificed two days after transplantation and single-cell suspensions obtained from the corpus callosum were visualized by an inverted fluorescence microscope (Olympus IX71, Japan). Nuclear staining was performed using DAPI to detect cells present in the corpus callosum.

### Cuprizonemouse model

A total of 24 male C57BL/6 mice were fed 0.2% (w/w) cuprizone (Sigma) in ground breeder chow,ad libitum for six weeks.This diet leads to selective oligodendrocyte death followed by demyelination of axons that are primarilylocated in the corpus callosum (11). At day 0 after cuprizone removal, animals were randomly divided into two groups: a. vehicle control group, which received 500 μl of DMEM alone (n=12) and b. transplant group, which received transplanted ADSCs (n=12). Quantities of 10×10^5^ ADSCs labeled with fluorescent dye (PKH26) in a volume of 500 μl of DMEM were transplanted into the lateral tail veinsusing a 27-gauge needle. At ten days after transplantation, animals from each group were killed and analyzed by flow cytometry, light and electron microscopy.The healthy control group (n=12) consisted of mice of the same age that were fed a normal diet (10).

All study procedures were conducted according to the Guidelines of the Animal Experiments of Research Council at Tehran University of Medical Sciences (Tehran, Iran).

### Single-cell suspension of corpus callosum

The complete corpus callosum was microdissected from PBS-perfused mice at two and ten days after ADSCs transplantation(9, 10). The tissue was placed into a Petri dish that contained 2 ml of digestion buffer, 1 mg/ml of collagenase D (Roche), 1 mg/ml of neutral protease (Worthington), and DNase I (Qiagen, Germany), and diced into small pieces with a razor blade before incubation at 37˚C for 30 minutes. Following incubation,we added PBS to stop the enzymatic digestion. Cells were washed through a 70 μm filter with FACS buffer, and then centrifuged at 2000 rpm for 5 minutes at 4˚C. The supernatant was aspirated and the pellet resuspended in 8 ml of 40% Percoll (Sigma) and layered onto 3 ml of 70% Percoll. The gradient was centrifuged at 2000 rpm for 25 minutes at room temperature without brakes. The cells were collected at the interface of the 40 and 70% Percoll and washed with PBS by centrifugation. Cells were pooled and transferred to a separate 15 ml tube, washed twice with PBS, and fixed in 4% formaldehyde (v/v in PBS) for 20 minutes on ice. The cells were then distributed between four microtubes per 15-ml tube and incubated with either anti-GFAP antibody (1:500, Millipore), anti-Iba-1 antibody (1:600, Millipore), anti- Olig2 antibody (1:100, Millipore) or anti-O4 antibody (1:50, Millipore) for 1 hour on ice. Cells were then washed three times with PBS, FITC-conjugated goat anti-rabbit IgG (1:100 dilution; Invitrogen, USA was added for 30 minutes on ice and the cells were washed three times and resuspended in PBS. The percentage of fluorescent cells was then analyzed using a FACSCalibur flow cytometer (BD Biosciences), with a total cell count of 10000 events (12).

### Preparation of brain tissue for histology

Ten days after ADSCs transplantation, mice were perfused with 4% PFA (Fluka) for demyelination staining,luxol fast blue (LFB), or 4% glutaraldehyde (GLA, Fluka) for resin embedding (n=3/ group). Healthy control mice were also perfused with 4% PFA or 4% GLA.

### Myelin staining

To stain for myelin content, tissue sections from mice were treated with LFB (Sigma, USA). Sections were stained overnight in LFB at 56˚C, then washed in 95% ethanol and distilled water to remove excess blue stain. The color was subsequently differentiated (until the white matter was easily distinguishable from the gray matter) in a lithium carbonate solution for 15 seconds, followed by distilled water and three washes of 80% alcohol. Slides were twice passed through fresh xylene, mounted with Entellan® (Merck, Germany), and cover slipped (1).

### Electron microscopic examination

Mice were transcardially perfused with 2% GLA and 2% PFA in 0.1 M PBS. Brains were removed, fixed with 1% osmium tetroxide and embedded in resin. Ultrathin sections were cut and stained with uranyl acetate and lead citrate, then observed with a transmission electron microscope (LEO 906 Germany, 100 kV). Images were taken from four sections, 50 μm apart, for each animal at locations between -0.94 mm and -1.28 mm from the bregma with the corpus callosum at the epicenter. Photographs of the axons cut in cross-section were taken and quantification of myelinated axons was performed on four, ×3000 and ×72500 images per animal, then analyzed with Image tools software Image J software. For morphometric analysis, we measured at least 100 axons. We counted the total numbers of axons with and without myelin sheaths contained within all of the selected electron micrographs from each animal. We measured the axonal diameter (d) as the shortest distance across the center of axons, avoiding the myelin sheath thickness. The axonal diameter plus the total myelin sheath thickness on both sides was defined as the fiber diameter (D). The G-ratio was calculated using the d/D ratio. Therefore, a completely demyelinated fiber would have a G-ratio=1, and in the myelinated fibers, the ratio would be <1. The Gratio was calculated for each myelinated fiber, and then the average from all the G-ratio values from one brain was calculated. The mean of the average G-ratio from the three brain samples were determined for each group (13).

### Statistical analysis

Statistical analysis was performed by one-way analysis of variance (ANOVA). Data was reported as means ± SEM. Each value represented the average of n=5 animals. Values of p<0.05 were considered statistically significant.

## Results

### Characterization of mouse adipose mesenchymal stem cells

**Fig 1:**
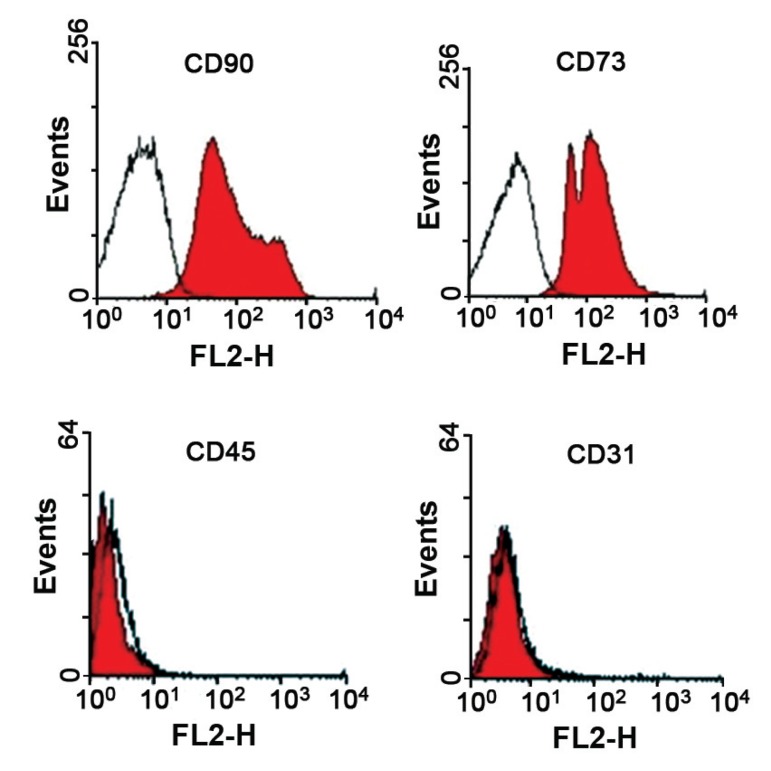
Flow cytometry analysis of C57BL/6 mice ADSCs showing that they do not express CD31, and CD45, but express CD73 and CD90. The white histograms show isotypematched control staining.

Flow cytometry analysis of passaged 3-5 mouse ADSCs showed that ADSCs were CD73^+^ (97%) and CD90^+^ (96%), but CD31- and CD45- (hematopoietic marker; Fig 1).

### Homing properties of adipose mesenchymal stem cells

**Fig 2:**
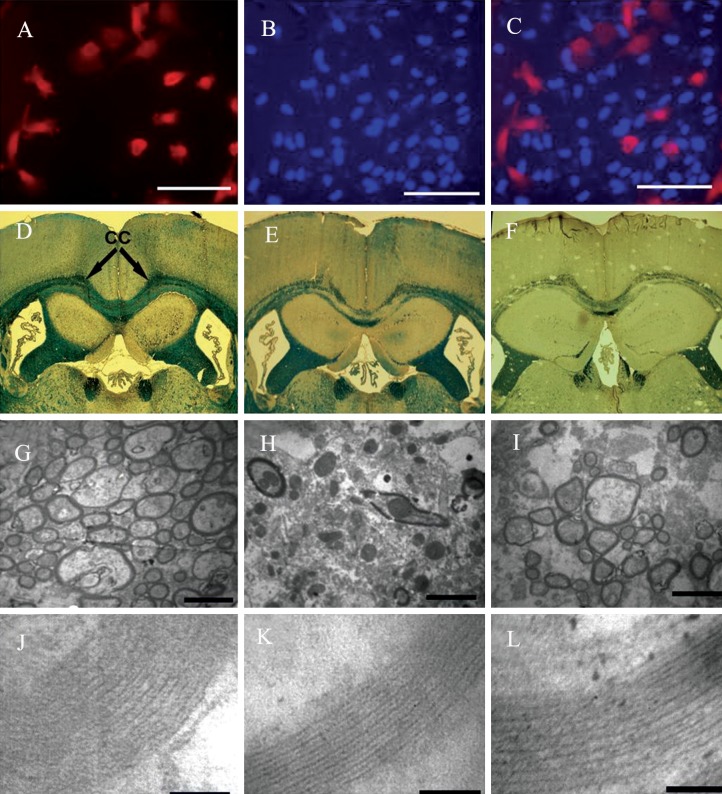
Fluorescence images (first row) of a pooled corpus callosum single-cell suspensionfrom recipient mice 2 days after transplantation show PKH26+adipose mesenchymal stem cells (ADSCs; red color), with DAPI bluestained nuclei, visualized among various cell types. ADSCs stained with PKH26 (A); nuclear staining with DAPI (B); and merge them (C). Light (second row) and transmission electron micrographs (third and fourth rows) showtransplantation of ADSCs facilitates remyelination in the corpus callosum of mice after cuprizone-induced demyelination.The photomicrographs were taken from coronal (light micrographs) and sagittal sections (transmission electron micrographs) of the corpus callosum of miceeuthanized 10 days after transplantation.(D-F): Myelin content evaluated by luxol fast blue (LFB) staining. Corpus callosumof a control health mouse (D, delineated by black lines); control vehicle (E); and transplanted group (F). (G-L): Electron micrographs show myelinated and unmyelinated axons at 10 days after treatment. Electron micrograph magnifications: ×3000, ×72500. (G, J) control health group; (H, K) control vehicle group; and (I, L) ADSCs transplantation group.Scale bars: A-C=100 μm; G-I=1 μm; and J-L=50 nm.

According to fluorescent cell labeling, the transplanted ADSCs appeared to survive and exhibited homing specificity. Fluorescent PKH26- labeled ADSCs that were intravenously transplanted localized almost exclusively in a singlecell suspension of the injuredcorpus callosum at two daysfollowing transplantation (Figs 2A-C).

### Light microscopic examination

Histologic examination of tissue sections was performed at ten days following ADSCs transplantation. LFB histologic stain was used to assess the extent of demyelination and remyelination. High intensity staining was evident in the healthy control group (Fig 2D).One week after transplantation of ADSCs an obvious remyelination was noted (Fig 2F); however the vehicle control group had less remyelination (Fig 2E).

### Improved myelination in adipose mesenchymal stem cells transplantation

**Fig 3:**
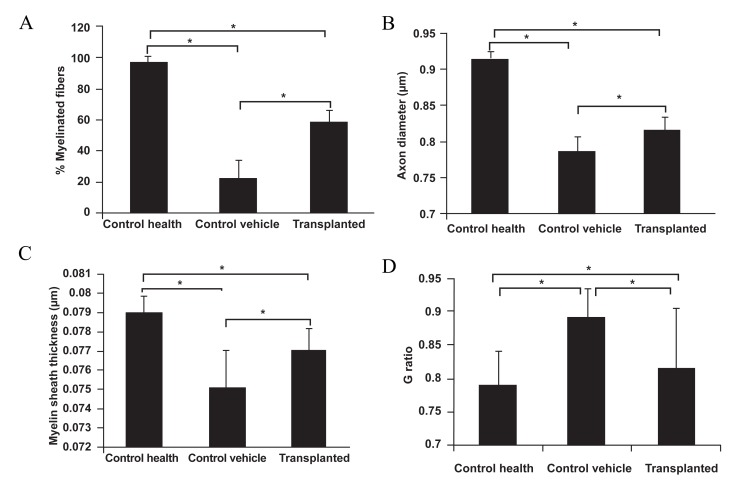
Percentage of myelinated axons in the corpus callosum (A); Mean of axon diameters (B); Mean of myelin sheath thickness (C); and G-ratio (D). Quantitative analysis of the electron micrographs was performedwith Image tools J software. Results are mean ± SEM off our different measurements for each experimental condition (*p<0.05).

Transmission electron microscopic photographs were used to determine myelin morphometric parameters from corpus callosum in the healthy control, vehicle control and transplanted groups (Fig 2G-L). Electron microscopicimages were taken from ultrathin sagittal sections obtained from the corpus callosum and analyzed to quantify the percentage of myelinated axons, axonal diameter, myelin thickness, and G-ratio (Fig 3). The results for the percentage of myelinated axons are presented in figure 3A. The axons in the corpus callosum were nearly completely demyelinated after six weeks of cuprizone feeding (not show). The mean myelin fibers significantly increased in the transplanted group (p≤0.05; 59 ± 8) compared with the vehicle control (p≤0.05; 22 ± 9) but was less than the healthy control group (p≤0.05; 98 ± 3; Fig 3A). The mean axonal diameter was significantly smaller in the transplanted group (p≤0.001; 0.815 ± 0.019 μm) compared with the healthy control group (0.935 ± 0.012 μm) but larger than the vehicle control group (p≤0.001; 0.784 ± 0.023 μm; Fig 3B). The myelin sheath was significantly thicker in the transplanted group (p≤0.001; 0.077 ± 0.006 μm) compared with the vehicle control group (p≤0.001; 0.075 ± 0.008 μm) but thinner than the healthy control group (p≤0.001; 0.079 ± 0.006 μm; Fig 3C). Correspondingly, the mean G-ratio significantly increased in the vehicle control (p≤0.001; 0.89 ± 0.045) compared with the healthy control group (p≤0.001; 0.78 ± 0.05). After ADSCs transplantation, the G-ratio recovered to an intermediate value (p≤0.05; 0.81 ± 0.09; Fig 3D).

### Short-term transplantation of ADSCs after demyelination changes in cellular composition of the corpus callosum

**Fig 4:**
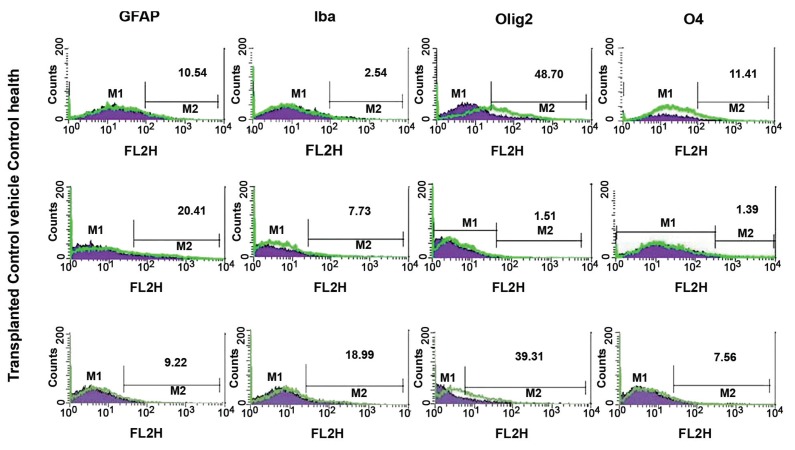
Analysisof changes in cellular composition in the corpus callosum of cuprizone-induced demyelinated mice treated with adipose mesenchymal stem cells (ADSCs) or vehicle alone. Mononuclear cells were isolated from the corpus callosum and the frequencies of GFAP+ (astrocytes), Iba-1 (microglia), Olig2^+^ (oligodendroglial progenitor) and O4^+^ (oligodendrocytes) cellswere determined by flow cytometryten days after transplantation. The respective isotype control is shown as a violet color.

The neural cell composition of cuprizone-induced demyelination lesions (corpus callosum) was altered in the presence of ADSCs (Fig 4). In the control vehicle group, the corpus callosum contained reduced proportions of Olig2^+^ and O4^+^ cells compared to the healthy control group.This loss significantly reversed in animals that received ADSCs. The proportion of Olig2^+^ cells in lesions increased from approximately 1.51% in the control vehicle group to 39.31% (p<0.05) in the ADSCs-transplanted group compared to approximately 48.70% in the healthy control group. Likewise, the number of O4^+^ oligodendrocytes increased from approximately 1.39 to 7.56% (p<0.05), which was closer to the healthy control value of 11.41%. The control vehicle group showed increased GFAP+ and Iba-1 +cells compared to the healthy control group. The proportion of GFAP+cells in the control vehicle group increased from approximately 10.54% in the control healthy group to 20.41% (p<0.05) in the control vehicle group. This increase significantly reversed in animals that received ADSCs (approximately 9.22%). The number of Iba-1 +cells increased from approximately 2.54% in the control healthy group to 7.73% in the control vehicle and 18.99% in ADSCs-transplanted groups.

## Discussion

MS is an inflammatory disease of the CNS characterized by extensive mononuclear cell infiltration and demyelination. MS is generally considered to be a T-cell mediated disease based on local inflammation (14), response to immune modulation or immunosuppression (15). The etiologyis unknown, but there is evidence for a role of both genetic and environmental factors (16). HLA genes are the most strongly associated genes in MS, and low vitamin D levels in serum, Epstein Bar infection, oxidative stress and smoking are currently the best documented environmental risk factors (17). The pathogenesis of MS is mainly driven by CNS-invading encephalitogenic CD4^+^ T lymphocytes of both the Th1 and Th17 types. These effectors cells can be down-regulated by regulatory T lymphocytes (18). The hallmarks of MS pathology include inflammation, demyelination, axonal loss, vascular abnormalities, iron accumulation, mitochondrial dysfunction and changes in cellular membrane permeability and sodium channels (16). In demyelinating diseases, remyelination and subsequent restoration of neuronal function can be achieved by either promoting endogenous repair mechanisms or by providing an exogenous source of myelinating cells via transplantation (19). However, the source and availability of stem cells is becoming a crucial issue for their clinical application (5). In this regard, several investigators have shown that BMSCs can remyelinate axons in an experimental autoimmune encephalomyelitis (EAE) model of MS (9, 20).To date, there has been one report of intravenous transplantation of ADSCs in an EAE model of MS (5).

In the present study, we have demonstrated that transplantation of homologous ADSCs can remyelinate demyelinated corpus callosum axons after intravenous transplantation in a cuprizone model of MS.Our results revealed intravenously transplantation ADSCs could migrate into the demyelinated lesion. Increasing clinical interest exists in the use of transplantable stem cells as a means of repairing neurodegenerative disease (9). The key to successful of such approaches will be dependent on the mode of delivery. Studies have begun to evaluate the clinical efficacy of using intravenously administered MSCs in diseases such as MS (5, 9, 10). Intravenously delivered cells are unlikely to migrate across the blood-brain barrier (BBB) into normal corpus callosum tissue because the BBB would prevent cell access to the parenchyma (21).

In this study PKH26-labeled ADSCs, transplanted via the lateral tail vein, were detected in singlecell suspension that was prepared fromthe corpus callosum. Jackson et al. (9) has shown that relatively minor, focal lesions provided sufficient cues to attract stem cells from the peripheral vasculature. The cuprizone model of MS induced a focal demyelination which might have provided the chemo attractant signals for mesenchymal stem cells such as ADSCs (22).

According to Ferrari et al. (23) intravenously transplanted bone marrow cells appear to be recruited through the vascular system and may be allowed to enter the lesion site because of the partial disruption of the BBB in a focal demyelination model. In addition, there may be active targeting mechanisms in the pathological environment of the lesion. Expression of chemotactic factors such as monocyte chemoattractant protein 1 (24) and matrix metalloproteases (MMPs) (25) are increased in the damaged CNS tissues. Adhesion molecules such as vascular adhesion molecule 1 and E-selectin are also highly expressed on the endothelial cells in the damaged lesions (26). In addition, Constantin et al. (5) report that the beneficial effect of ADSCs on chronicdemyelination relies also on the ability to penetrate into the CNS due to the expression on a significant ADSC subset of activated α4 integrin, a key adhesion molecule involved inleukocyte and stem cell migration into the inflamed CNS.Thus, the interaction of these molecules may promote the transplanted ADSCs to target the corpus callosum lesions.

Our data showed that administration of ADSCs into amousecuprizone model of MS resulted in benefits confirmed by histological analyses. These results supported results of previous studies that suggested a therapeutic benefit of MSCs transplantation for the cure of neurodegenerative disease such as MS (3, 5, 9, 20).

Although the cellular mechanisms responsible for the therapeutic effects of ADSCs on remyelination remain unclear, two hypotheses should be considered. ADSCs as MSCsmay participate inremyelination by either differentiating into mature oligodendrocytes that can form new myelin or indirectly by promoting the survival and proliferation of endogenous precursor cells (5). The possibility that MSCs benefit cerebral tissue by becoming brain cells is very unlikely. With intravenous injection numbering a few hundred thousand at most, there are very few cells present (even if they become brain cells) to replace a volume of tissue of more than a few cubic millimeters (26). Thus, replace damaged tissue as the mechanism by which ADSCs promote their beneficial effects is very unlikely (27). A far more reasonable explanation is that MSCs induce cerebral tissue to activate endogenous restorative effects of the brain (1). MSCs may turn on reactions and interact with the brain to activate restorative and possibly regenerative mechanisms (28).

Constantin et al. have shown that ADSCs are able to secrete basic fibroblast growth factor (bFGF), brain-derived growth factor (BDNF) and platelet-derived growth factor (PDGF), all which strongly support the process of oligodendrogenic differentiation (5). Our results have shown that ADSCs induced improvement was accompanied by changes in neural cell composition with increased microglia and decreased astrocytes in the lesion areas.In similar experiments, Short et al. (12), Eglitis and Mezey (29) and Tambuyzer et al. (30) also reported that the numbers of microglial cells increased in the demyelination lesion after heterologous MSCs transplantation. Tambuyzer et al. (30) have suggested that microglia is able to discriminate between self and non-self *in vivo*; although a clear mechanism for the latter still remains to be elucidated. A potential mechanism for clearing allograft in the CNS by microglia maybe found in their production of TNF-α and/or NO on strong activation (30, 31). It has recently been demonstrated that interleukin-17-producing T cells (ThIL-17) mediate inflammatory pathology in certain autoimmune diseases, including MS (32). Previous studies suggest that astrocytes can stimulate IL-17 production (33). Treatment with MSCs significantly down regulates IL-17 levels and results in a reduction in astrogliosis (16).

## Conclusion

These results support the use of appropriate ADSCs as useful tools for translational studies on demyelinating neuronal disorders. They provide a reinforcing element when considering the therapeutic role of ADSCs for the cure of neurodegenerative diseases such as MS that are characterized by pathological hallmarks such as demyelination. In addition, our results indicate that intravenous injection is an ideal minimally invasive technique to deliver cellular transplants to the injured brain.
